# Treatment patterns and clinical outcomes of chidamide combined with endocrine therapy in hormone receptor‐positive, HER2‐negative metastatic breast cancer: A real‐world multicenter study

**DOI:** 10.1002/cam4.6762

**Published:** 2024-03-08

**Authors:** Doudou Li, Yizi Jin, Mingxi Lin, Cheng Zeng, Qing Guo, Yanfei Liu, Jian Zhang

**Affiliations:** ^1^ Department of Medical Oncology Fudan University Shanghai Cancer Center Shanghai China; ^2^ Department of Oncology Shanghai Medical College, Fudan University Shanghai China; ^3^ MN. Office of Clinical Research Fudan University Shanghai Cancer Center Shanghai China; ^4^ Phase I Clinical Trial Center Fudan University Shanghai Cancer Center Shanghai China

**Keywords:** chidamide, endocrine therapy, hormone receptor‐positive, metastatic breast cancer, real‐world data

## Abstract

**Background:**

Chidamide is a selective histone deacetylase inhibitor approved for patients with hormone receptor (HoR)‐positive and HER2‐negative metastatic breast cancer (MBC). We aimed to investigate the efficacy, safety, and treatment patterns of chidamide and identify clinicopathological factors that predict the efficacy of chidamide in real‐world scenarios.

**Methods:**

Consecutive MBC patients treated with chidamide from January 2020 to August 2021 across 11 institutions were enrolled in this multicenter, retrospective study. Eligible patients were pre‐ and postmenopausal women who had clinically or histologically confirmed ER‐positive, HER2‐negative MBC, and Eastern Cooperative Oncology Group (ECOG) performance status of 0 or 1. Patients with multiple primary malignancies or missing baseline characteristics were excluded. Patients received 30 mg chidamide orally twice a week, combined with aromatase inhibitors (AIs) or non‐AIs. Efficacy analyses included progression‐free survival (PFS), objective response rate (ORR), and clinical benefit rate (CBR). Univariate and multivariate Cox regression analyses were performed to identify the potential efficacy predictors.

**Results:**

A total of 157 patients were finally included for analysis. The median number of lines prior to chidamide was four. In the whole cohort, the median PFS was 4.2 months (95% confidence interval [CI] 3.8–4.5). The ORR was 7.5% and the CBR was 31.3%. The efficacy of chidamide was consistent in patients pretreated with CDK4/6 inhibitors and patients treated with different endocrine combinations. Multivariate analysis indicated that patients who had liver metastases (adjusted HR = 1.66, 95% CI 1.14–2.43, adjusted *p* = 0.008) or ≥3 prior lines of treatment (adjusted HR = 1.80, 95% CI 1.17–2.77, adjusted *p* = 0.008) had significantly worse PFS. The most common AEs with chidamide were thrombocytopenia, leucopenia, neutropenia, and anemia.

**Conclusion:**

This study provided real‐world data for the use of chidamide in patients with HoR‐positive and HER2‐negative MBC. Our data endorsed the use of chidamide in patients pretreated with CDK4/6 inhibitors and patients treated with different endocrine combinations.

## 1 INTRODUCTION

Breast cancer has become the most frequently diagnosed malignancy worldwide,^1^ and hormone receptor (HoR)‐positive breast cancer represents the majority of breast cancer patients.^2^, ^3^ Endocrine therapy suppresses tumor growth and development through blocking estrogen/estrogen receptor (ER) axis. It greatly reduces cancer recurrence and prolongs patients' survival and is the mainstay of HoR‐positive breast cancer treatment. However, the efficacy of endocrine therapy is considerably challenged by intrinsic or acquired endocrine resistance that can occur in a large subset of HoR positive patients.^4^ Even though the advent of cyclin dependent kinase (CDK) 4/6 inhibitors (e.g., palbociclib, abemaciclib, ribociclib, and dalpiciclib) has marked a new era of HoR‐positive breast cancer treatment, the addition of CDK4/6 inhibitors can only abrogate part of endocrine resistance. Treatment choices after disease progression on CDK4/6 inhibitors remain limited. Therefore, it is significant to explore the mechanisms and develop more effective strategies to overcome endocrine resistance in HoR‐positive patients, especially those with recurrent or metastatic breast cancer (MBC).

Chidamide (also known as tucidinostat) is an oral type, selective histone deacetylase (HDAC) inhibitor, which acts through epigenetic regulations to induce cell cycle arrest and apoptosis in tumor cells. Epigenetic reprogramming impacts ER downstream transcription factors, triggers parallel pathways independent of estrogen/ER that promote tumor growth, and is implicated with endocrine resistance. Preclinical studies have shown the potential efficacy of HDAC inhibitors to enhance the sensitivity of AIs and suppress aberrantly activated growth factor signaling pathways in breast cancer.^5^, ^6^ Chidamide has also been reported to enhance antibody‐dependent cell‐mediated cytotoxicity and cytotoxic T cell‐mediated anti‐tumor immunity,^7^, ^8^ and reverse epithelial‐mesenchymal phenotype transformation (EMT) of tumor cells.^9^ In an exploratory phase II trial, chidamide plus exemestane was adequately tolerated and effective in patients with HoR‐positive MBC.^10^ A phase III randomized controlled trial (ACE study) proved that chidamide combined with exemestane improved progression‐free survival (PFS) in patients with advanced, HoR‐positive breast cancer who progressed after previous endocrine therapy compared with placebo plus exemestane.^11^ Based on the evidence from ACE trial, chidamide was approved for postmenopausal patients with HoR‐positive, human epidermal growth factor receptor 2 (HER2)‐negative advanced or MBC who relapsed or progressed on endocrine therapy.

Further investigations on its efficacy and safety in the real‐world clinical practice is still warranted. In the ACE trial, only very few patients received CDK4/6 inhibitors since none of these agents were approved in China during the recruitment of the ACE study. However, in real world, a large proportion of metastatic patients had the treatment with CDK4/6 inhibitors in the frontier lines since it has been set as new standard. Therefore, the benefit of chidamide in patients who progressed on CDK4/6 inhibitors needs further investigation. Additionally, over three quarters of the patients treated with chidamide received <2 lines of previous treatments for MBC in the trial, while patients treated with chidamide in real‐world scenario tend to have more prior lines of therapy. Furthermore, more data on the combination of chidamide with other anti‐estrogen agents are needed since many patients have been pretreated with exemestane in daily practice. In this multicenter study, we aimed to investigate the real‐world effectiveness, toxicity, and treatment patterns of chidamide in patients with HoR‐positive MBC and find potential clinicopathological factors for efficacy prediction.

## 2 PATIENTS AND METHODS

### 2.1 Study population and treatment

This multicenter cohort study enrolled consecutive breast cancer patients treated with chidamide from January 2020 to August 2021 across 11 institutions, including Fudan University Shanghai Cancer Center, Zhongshan hospital, Huashan hospital, Huadong hospital, Shanghai East Hospital, Shanghai Tenth People's Hospital, Changhai Hospital, Shanghai General Hospital, Renji Hospital, Ruijin Hospital, and Huangpu Branch of Shanghai Ninth People's Hospital. All data were retrospectively collected through the institutions' electronic medical record. Eligible patients were pre‐ and postmenopausal women with clinically or histologically confirmed ER‐positive, HER2‐negative breast cancer, who had at least one recurrent/metastatic lesion; and Eastern Cooperative Oncology Group (ECOG) performance status of 0 or 1. Patients with multiple primary malignancies or unavailable baseline characteristics were excluded.

Patients received 30 mg chidamide orally twice a week for four consecutive weeks in a 4‐week cycle, combined with exemestane 25 mg orally daily/anastrozole 1 mg orally daily/letrozole 2.5 mg orally daily/tamoxifen 10 mg orally twice daily/toremifene 60 mg orally daily/fulvestrant 500 mg intramuscularly once a month. Each premenopausal patient received ovarian function suppression (OFS) with concurrent endocrine therapy. Treatment was administered until disease progression, intolerable toxicity, or patient refusal.

### 2.2 Outcome

Efficacy analyses included PFS, objective response rate (ORR), and clinical benefit rate (CBR). PFS was defined as the time from treatment initiation of chidamide to disease progression or death due to any cause. ORR was defined as the proportion of patients achieved complete response (CR) or partial response (PR) as the best objective tumor response. CBR was defined as the proportion of patients who achieved CR, PR, or had stable disease (SD) for at least 24 weeks as the best objective tumor response. Tumor assessments were performed approximately every 3 months or when signs of disease progression were shown until treatment discontinuations. Efficacy was assessed based on regular CT and/or MRI tests according to the Response Evaluation Criteria in Solid tumors (RECIST) 1.1.^12^ Adverse events (AEs) and laboratory abnormalities were documented every regular follow‐up according to the National Cancer Institute Common Terminology Criteria for Adverse Events (NCI‐CTCAEs) version 5.0.

### 2.3 Statistical analyses

Efficacy analyses and safety analyses were performed in all included patients. We calculated median PFS and plotted time‐to‐event curves using Kaplan–Meier method, and compared the median PFS of different subgroups using log‐rank tests. Hazard ratios (HRs) with 95% confidence intervals (CIs) were calculated with the Cox proportional hazards model. Univariate analyses were performed to screen the potential variables associated with PFS, including menstrual status (premenopausal vs. postmenopausal), age (<65 vs. ≥65 years old), ECOG status (0 vs. 1), visceral metastases (yes vs. no), lung metastases (yes vs. no), liver metastases (yes vs. no), number of metastatic organs (0–1 vs. ≥2), number of lines prior to chidamide (0–2 vs. ≥3), previous endocrine therapy for MBC (yes vs. no), sensitive to endocrine therapy (yes vs. no), previous use of CDK4/6 inhibitors (yes vs. no), previous chemotherapy for MBC (yes vs. no). Sensitivity to endocrine therapy was defined as at least 24 months of endocrine therapy before recurrence in the adjuvant setting or a response or stabilization for at least 6 months with endocrine therapy for advanced disease. In patients who relapsed while on adjuvant endocrine therapy or within 12 months of completing adjuvant endocrine therapy, adjuvant endocrine therapy was considered as an additional line prior to the treatment with chidamide. Variables significant in the univariate analyses were further evaluated in the multivariate analyses. Multivariable cox proportional hazards models with forward selection were used to identify independent predictors of PFS. All *p*‐values were two‐sided and considered statistically significant when <0.05.

## 3 RESULTS

### 3.1 Patient characteristics

**FIGURE 1:**
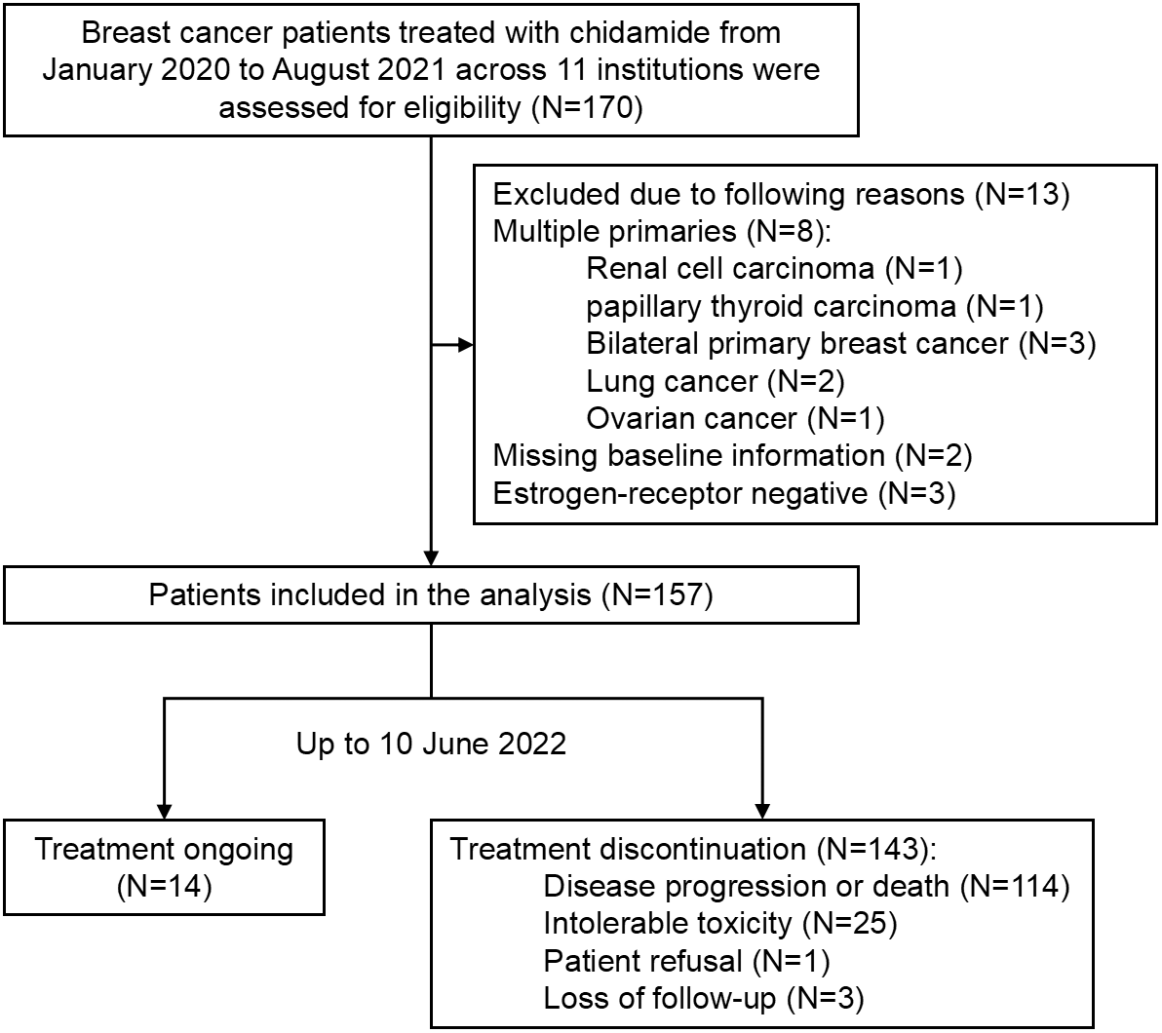
Flow chart of patient selection.

From January 2020 to August 2021, 170 breast cancer patients treated with chidamide were assessed for eligibility and 157 patients were finally included for analysis. Reasons for exclusion have been detailed in the patient selection flow chart (Figure 1). Clinicopathological characteristics and treatment combinations of included patients are reported in Table 1. The median age of included patients was 55 years old, and most patients (80.9%) were postmenopausal. 126 patients (80.3%) were both ER‐ and PR‐positive, while 19.7% patients were PR‐negative. Most of the patients (70.1%) had visceral metastases, including lung (43.9%), brain (3.8%), and liver (43.3%). Bone involvement occurred in 70.1% of all included patients.

**TABLE 1:** Baseline characteristics.

Characteristics	Patients (*N* = 157)	%
Age (years)		
Mean ± SD	56.67 ± 10.57	
Median	55	
Range	33–86	
Menstrual status		
Premenopausal	30	19.1%
Postmenopausal	127	80.9%
ECOG status		
0	3	1.9%
1	154	98.1%
HoR status		
ER‐positive/PR‐positive	126	80.3%
ER‐positive/PR‐negative	31	19.7%
Metastatic site		
Visceral	110	70.1%
Lung	69	43.9%
Brain	6	3.8%
Liver	68	43.3%
Bone	110	70.1%
Number of metastatic organs^a^		
<2	68	43.3%
≥2	89	56.7%
Number of lines prior to chidamide^b^		
Median	4	
Range	0–13	
0	2	1.3%
1	11	7.0%
2	33	21.0%
3	28	17.8%
4 and more than 4	83	52.9%
Previous endocrine therapy for metastatic disease		
Yes	137	87.3%
No	20	12.7%
Previous use of CDK4/6 inhibitors		
Yes	86	54.8%
No	71	45.2%
Sensitive to previous endocrine therapy^c^		
Yes	110	70.1%
No	47	29.9%
Previous chemotherapy		
For neoadjuvant treatment	7	4.5%
For adjuvant treatment	123	78.3%
For metastatic settings	95	60.5%
Combination therapy		
Aromatase inhibitors	112	71.3%
Fulvestrant	14	8.9%
Selective estrogen receptor modulators	12	7.6%
Unknown	19	12.1%

Abbreviations: ECOG, Eastern Cooperative Oncology Group; ER, estrogen receptor; HoR, hormone receptor; PR, progesterone receptor; SD, standard deviation.

^a^ Only visceral and bone metastases were calculated.

^b^ In patients who relapsed while on adjuvant endocrine therapy or within 12 months of completing adjuvant endocrine therapy, adjuvant endocrine therapy was considered as an additional line prior to the treatment with chidamide.

^c^ Sensitivity was defined as at least 24 months of endocrine therapy before recurrence in the adjuvant setting or a response or stabilization for at least 6 months with endocrine therapy for advanced disease.

The median number of lines prior to chidamide was four (ranging from 0 to 13). Only two patients received chidamide as the first‐line therapy, and 11 patients received one prior line of therapy before chidamide. A vast majority of patients (87.3%) had previous endocrine therapy and most patients (60.5%) had previous chemotherapy for metastatic disease. Over a half of patients (54.8%) received endocrine therapy combined with CDK4/6 inhibitors previously. 47 patients (29.9%) had endocrine resistance and 110 patients (70.1%) were sensitive to previous endocrine therapy. Chidamide were most commonly combined with aromatase inhibitors (AIs) (71.3%), while fulvestrant (8.9%) and selective estrogen receptor modulators (SERMs) (7.6%) were also used in some patients.

Up to June 10, 2022, 14 patients (8.9%) were still receiving chidamide. Patients discontinued the treatment due to the following reasons: disease progression or death (114/157, 72.6%), intolerable toxicity (25/157, 15.9%), patient refusal because of additional comorbidities (1/157, 0.6%), or loss of follow‐up (3/157, 1.9%).

### 3.2 Efficacy analyses

**FIGURE 2:**
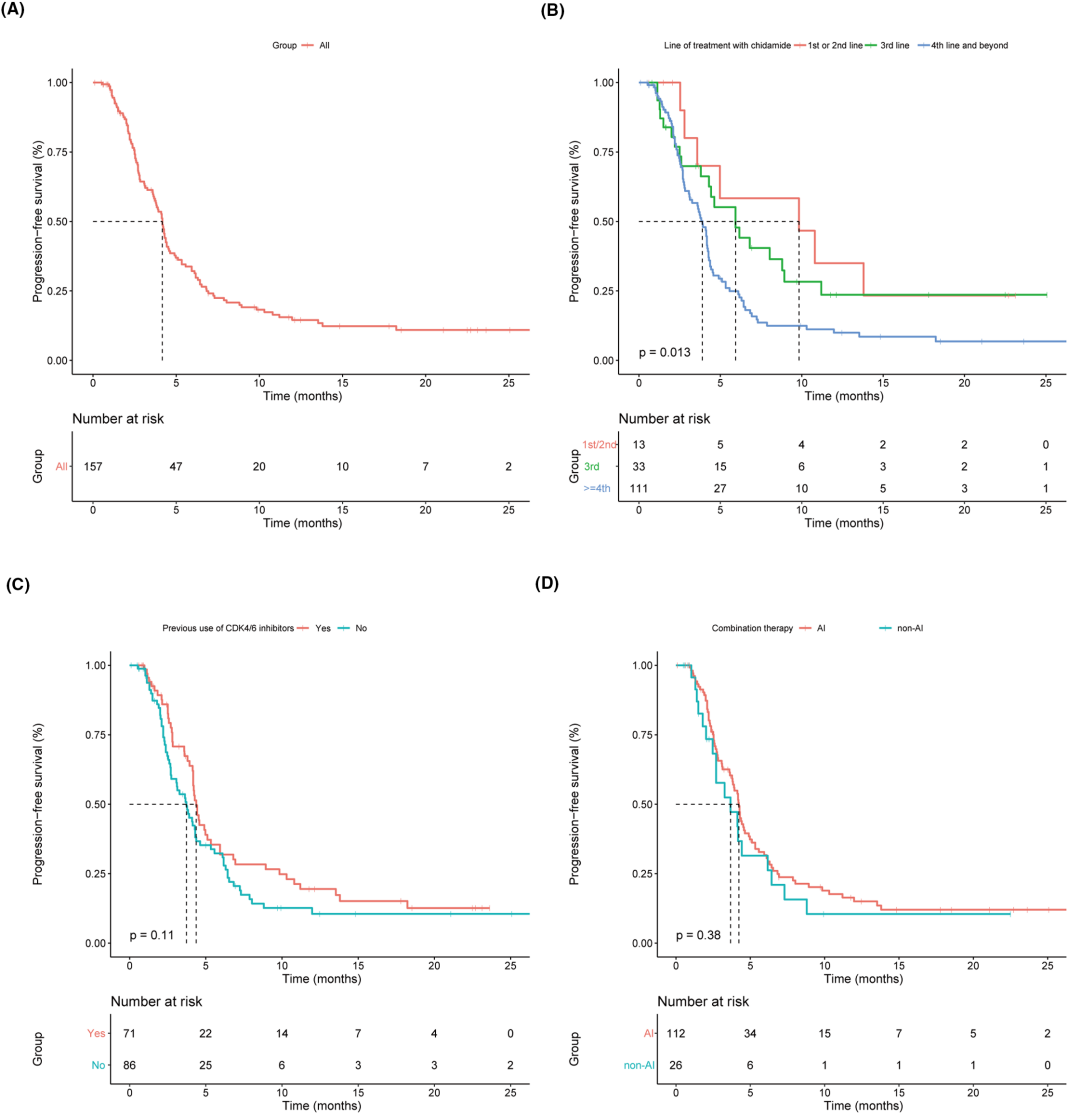
Kaplan–Meier plots for PFS in patients treated with chidamide. (A) PFS in all included patients; (B) PFS in patients stratified by line of treatment with chidamide; (C) PFS in patients stratified by previous use of CDK4/6i; (D) PFS in patients stratified by combination therapies. CDK4/6i, cyclin dependent kinase 4/6 inhibitors; PFS, progression‐free survival.

The median duration of follow‐up was 18.9 months (interquartile range [IQR] 14.8–23.4) at the cutoff date (June 10, 2022). In the whole cohort, the median PFS was 4.2 months (95% CI 3.8–4.5) (Figure 2A and Table 2). In evaluable patients, the ORR was 7.5% (10/134) and the CBR was 31.3% (42/134) (Table 2).

**TABLE 2:** Efficacy analysis.

	Number of patients (*N* = 157)
Progression‐free survival	
Disease progression or death	114 (72.6%)
Kaplan–Meier median, months	4.2 (3.8–4.5)
Best overall response	
Complete response	0
Partial response	10 (6.4%)
Stable disease	49 (31.2%)
Progressive disease	75 (47.8%)
Not evaluable or unknown	23 (14.6%)
Objective response^a^	10 (7.5%)
Clinical benefit^a^	42 (31.3%)

^a^ In evaluable patients.

*Note*: Data are *n* (%), median (95% CI), or *n*/*N* (%).

In patients treated with chidamide as the 1st‐/2nd‐line therapy, the median PFS was 9.8 months (95% CI 1.7–18.0). The median PFS was 5.9 months (95% CI 3.4–8.5) in patients received third‐line chidamide and was 3.9 months (95% CI 3.4–4.4) in patients who received chidamide in the fourth‐line or beyond settings (Figure 2B). The ORR was 15.4% (6/39) and the CBR was 46.2% (18/39) in evaluable patients treated with chidamide as the first−/second−/third‐line therapy.

Patients who had prior use of CDK4/6 inhibitors had a median PFS of 3.7 months (95% CI 2.8–4.6) and those who had not been treated with CDK4/6 inhibitors had a median PFS of 4.4 months (95% CI 4.0–4.7). The prior use of CDK4/6 inhibitors did not significantly affect PFS in patients treated with chidamide (*p* = 0.115) (Figure 2C). The median PFS of patients treated with chidamide combined with AIs (4.2 months, 95% CI 3.8–4.6) was comparable to those who combined with non‐AIs (3.7 months, 95% CI 1.7–5.7, *p* = 0.377) (Figure 2D).

Univariate Cox regression analyses demonstrated that liver metastases (*p* = 0.004), number of lines prior to chidamide (*p* = 0.004), previous endocrine therapy for MBC (*p* = 0.008), and previous chemotherapy for MBC (*p* = 0.016) were significantly correlated with PFS in patients treated with chidamide. The above variables significant in the univariate analyses were further evaluated in the multivariate analyses. We used multivariable cox proportional hazards model with forward selection to identify independent predictors of PFS. The variables included in the final multivariable cox proportional hazards model were liver metastases and lines of therapy. Multivariate analysis indicated that patients who had liver metastases (adjusted HR = 1.66, 95% CI 1.14–2.43, adjusted *p* = 0.008) or ≥3 prior lines of treatment (adjusted HR = 1.80, 95% CI 1.17–2.77, adjusted *p* = 0.008) had significantly worse PFS (Table 3).

**TABLE 3:** Univariate and multivariate analyses of progression‐free survival in patients treated with chidamide.

Characteristics	Univariate analysis			Multivariate analysis		
	HR	95% CI	*p*‐value	HR	95% CI	*p*‐value
Menstrual status						
Premenopausal	Reference					
Postmenopausal	1.44	0.90–2.31	0.126			
Age						
<65	Reference					
≥65	0.86	0.55–1.34	0.493			
ECOG status						
0	Reference					
1	0.72	0.23–2.26	0.567			
Progesterone‐receptor status						
Negative	Reference					
Positive	0.85	0.54–1.32	0.468			
Visceral metastases						
No	Reference					
Yes	1.38	0.92–2.08	0.119			
Lung metastases						
No	Reference					
Yes	0.98	0.67–1.42	0.910			
Liver metastases						
No	Reference			Reference		
Yes	1.74	1.19–2.53	0.004**	1.66	1.14–2.43	0.008**
Number of metastatic organs^a^						
<2	Reference					
≥2	1.38	0.95–2.01	0.090			
Number of lines prior to chidamide^b^						
0–2	Reference			Reference		
≥3	1.87	1.22–2.88	0.004**	1.80	1.17–2.77	0.008**
Previous endocrine therapy for metastatic disease						
No	Reference					
Yes	2.33	1.24–4.35	0.008**			
Sensitive to previous endocrine therapy^c^						
Yes	Reference					
No	1.33	0.89–1.98	0.159			
Previous use of CDK4/6 inhibitors						
No	Reference					
Yes	1.35	0.93–1.96	0.117			
Previous chemotherapy for metastatic disease						
No	Reference					
Yes	1.62	1.09–2.39	0.016*			

Abbreviations: CI, confidence interval; ECOG, Eastern Cooperative Oncology Group; HR, hazard ratio.

^a^ Only visceral and bone metastases were calculated.

^b^ In patients who relapsed while on adjuvant endocrine therapy or within 12 months of completing adjuvant endocrine therapy, adjuvant endocrine therapy was considered as an additional line prior to the treatment with chidamide.

^c^ Sensitivity was defined as at least 24 months of endocrine therapy before recurrence in the adjuvant setting or a response or stabilization for at least 6 months with endocrine therapy for advanced disease.

*p*‐values were considered statistically significant when <0.05 (in bold). *p <0.05; **p <0.01.

### 3.3 Treatment choices of next‐line therapy

**FIGURE 3:**
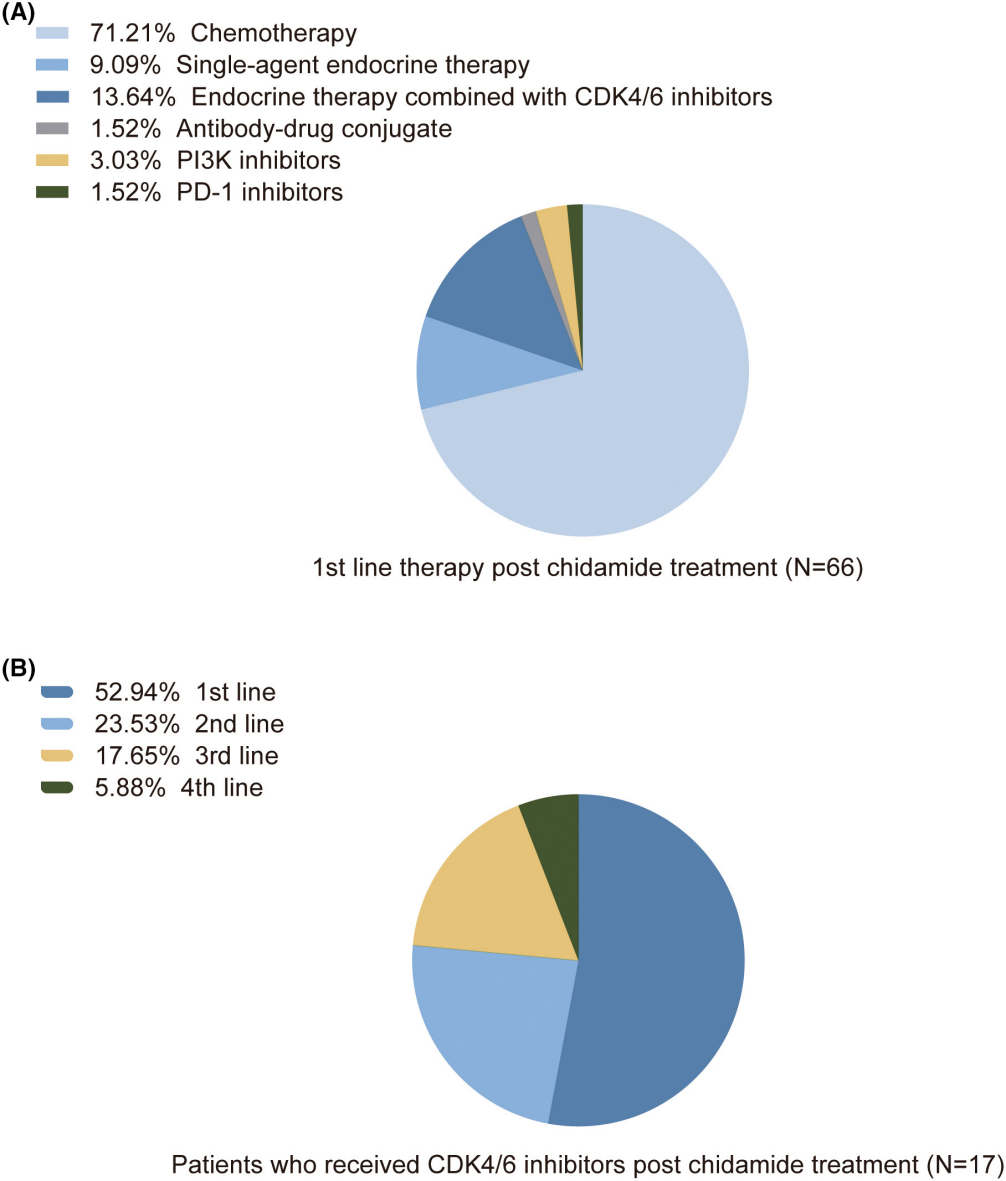
Treatment choices after chidamide treatment. (A) Next‐line treatment after chidamide treatment. (B) The proportion of patients treated with post‐first/second/third/fourth‐line CDK4/6 inhibitors. PD‐1, programmed cell death protein‐1.

Among the 66 patients with known next‐line therapy after chidamide treatment, 47 patients switched to chemotherapy as the post first‐line treatment, and 15 patients received subsequent endocrine therapy. A patient with HER2‐low breast cancer who progressed on chidamide was enrolled in the clinical trial and treated with trastuzumab deruxtecan (T‐DXd). Other post first‐line treatments included PI3K inhibitors and programmed cell death protein‐1 (PD‐1) inhibitors (Figure 3A). In the 17 patients who received endocrine therapy combined with CDK4/6 inhibitors post chidamide, most patients used CDK4/6 inhibitors in the post first‐line settings (Figure 3B).

### 3.4 Safety

The most common AEs with chidamide were thrombocytopenia (35.0%), leucopenia (26.8%), neutropenia (23.6%), and anemia (14.0%). Nausea (13.4%) and vomiting (10.2%) were also non‐hematological toxicities commonly seen in patients. The most common Grade 3/4 AEs were thrombocytopenia (10.8%) and neutropenia (6.4%). The hematological AEs were mostly manageable, and were reversible after the discontinuation of chidamide (Table 4). No cases of febrile neutropenia were reported.

**TABLE 4:** Adverse events.

Adverse events	Grade 1	Grade 2	Grade 3	Grade 4	All grade	(%)	Grade 3/4	(%)
Leucopenia	22	12	8	0	42	26.8%	8	5.1%
Neutropenia	14	13	9	1	37	23.6%	10	6.4%
Anemia	14	2	6	0	22	14.0%	6	3.8%
Thrombocytopenia	24	14	13	4	55	35.0%	17	10.8%
Increased aspartate/alanine aminotransferase	7	5	3	0	15	9.6%	3	1.9%
Increase blood creatine phosphokinase	1	0	1	0	2	1.3%	1	0.6%
Nausea	14	3	4	0	21	13.4%	4	2.5%
Vomiting	9	5	2	0	16	10.2%	2	1.3%
Diarrhea	7	4	1	0	12	7.6%	1	0.6%
Abdominal pain/abdominal distension	1	3	0	0	4	2.5%	0	0.0%
Anorexia	7	2	1	0	10	6.4%	1	0.6%
Fatigue	9	2	2	0	13	8.3%	2	1.3%
Pedal edema	1	2	1	1	5	3.2%	2	1.3%
Interstitial lung Disease	2	2	1	0	5	3.2%	1	0.6%
Pain	6	3	1	0	10	6.4%	1	0.6%
Thromboembolic Events	0	1	0	0	0	0.0%	0	0.0%
Dizziness	2	0	0	0	0	0.0%	0	0.0%
Palpitations	1	0	0	0	0	0.0%	0	0.0%

*Note*: For any listed adverse event, each patient was counted according to the worst severity grade.

## 4 DISCUSSION

In this multicenter, retrospective study, patients with HoR‐positive, HER2‐negative MBC receiving chidamide demonstrated a median PFS of 4.2 months. The ORR was 7.5% and the CBR was 31.3% in evaluable patients. The median PFS was 9.8 months in patients treated with chidamide as the first−/second‐line therapy and was 5.9 months in patients received third‐line chidamide. The ORR was 15.4% and the CBR was 46.2% in patients treated with chidamide as the first−/second−/third‐line therapy. The toxicity of chidamide was generally tolerable. The most frequently reported AEs were hematological toxicities, including thrombocytopenia, leucopenia, neutropenia, and anemia.

In the ACE study, the median PFS was 7.4 months (95% CI 5.5–9.2) in the chidamide group. The ORR was 18% and the CBR was 47% in patients treated chidamide in the clinical trial. The numerical difference in the response rate can be partly explained by the difference in treatment population. The ORR and CBR in the clinical trial were similar to that in patients treated with first−/second−/third‐line chidamide in this retrospective study. In the real‐world clinical practice, chidamide tend to be used in more heavily pretreated patients. The median number of lines prior to chidamide was four and the proportion of patients treated with previous 0–1 lines of therapy was 8.3% in this study. In contrast, in the ACE trial, over quarters of chidamide group received 0–1 lines of previous treatment for MBC and only 3% patients had three lines of previous therapy before chidamide in the metastatic settings. In addition, a higher proportion of patients in this study had previous chemotherapy and endocrine therapy for metastatic disease in this study (60.5% and 87.3%, respectively) compared to that in the clinical trial (30.0% and 47.1%, respectively). The proportion of patients with visceral disease was also higher than that in the trial. This study demonstrated antitumor effectiveness of chidamide even used in patients with higher tumor burden or as later lines of therapy.

This study provided evidence for scenarios that have not been adequately investigated in previous trials but commonly seen in clinical practice. Our data endorsed the use of chidamide in patients pretreated with CDK4/6 inhibitors. Based on the satisfactory efficacy of palbociclib,^13^, ^14^, ^15^ ribociclib,^16^, ^17^, ^18^, ^19^, ^20^, ^21^ abemaciclib,^22^, ^23^, ^24^ and dalpiciclib,^25^ CDK4/6 inhibitors combined endocrine therapy has been recommended as the first‐line treatment for patients with HoR‐positive, HER2‐negative MBC after progression on single‐agent endocrine therapy. Consequently, a large proportion of patients have used CDK4/6 inhibitors in the metastatic settings prior to chidamide in this study. Another multicenter study indicated that chidamide plus exemestane was one of the most frequently used endocrine therapy regimens in HoR‐positive, HER2‐negative MBC after progression on CDK4/6 inhibitors.^26^ The above evidence represents the real‐world treatment pattern for HoR‐positive, HER2‐negative MBC. Meanwhile, only very few patients had been previously treated with CDK4/6 inhibitors since none of these agents were approved in China during the recruitment of the ACE clinical trial. The real‐world efficacy data for this subpopulation in our study supplemented the previous findings. Univariate Cox regression analysis proved that the efficacy of chidamide was not affected by prior use of CDK4/6 inhibitors, lending support to the use of chidamide as a reasonable choice for those who progressed on treatment with CDK4/6 inhibitors.

Chidamide was approved in China for postmenopausal patients with HoR‐positive MBC, combined with AIs. Most patients in this study used chidamide plus AIs, while a small proportion of patients received fulvestrant or SERMs as combination regimens. Our data did not show significant difference in PFS between combinational AIs and non‐AIs when treated with chidamide. This finding indicated that those who previously progressed on AIs can also choose non‐AIs as combinational therapy without affecting efficacy. In premenopausal patients, this study proved that the efficacy of chidamide was comparable to that in the postmenopausal with simultaneous OFS.

Liver metastases (*p* = 0.004), number of lines prior to chidamide (*p* = 0.004), previous endocrine therapy for MBC (*p* = 0.008), and previous chemotherapy for MBC (*p* = 0.016) were found to be significantly associated with PFS in univariate analyses, while only liver metastases and prior lines of chidamide were identified as the independent predictors in multivariate analysis. Patients who had liver metastases (adjusted HR = 1.66, 95% CI 1.14–2.43, adjusted *p* = 0.008) or ≥3 prior lines of treatment (adjusted HR = 1.80, 95% CI 1.17–2.77, adjusted *p* = 0.008) had significantly worse PFS. This finding may help in patient selection and treatment planning. In addition, our analyses suggested that chidamide can be considered in fronter lines of therapy in suitable metastatic cases to achieve better clinical outcomes. Univariate analyses also indicated that chidamide was equally effective in patients with endocrine resistance. In daily practice, chemotherapy is sometimes preferred over endocrine therapy even as initial palliative treatment for MBC, especially for patients who are intrinsically resistant to endocrine therapy, or those who have life‐threatening visceral metastases.^27^, ^28^ This study suggested that chidamide combined with endocrine therapy was a reasonable choice for these patients who were stable after the completion of chemotherapy.

Hematological toxicity and gastrointestinal toxicity were the most commonly observed AEs in this study, which was consistent with the toxicity profile of chidamide and other HDAC inhibitors in previous studies.^29^, ^30^, ^31^ The hematological AEs were generally manageable and reversible. Rare AEs that have not been discussed in the previous trials included interstitial lung disease, thromboembolic events, and palpitations. The frequency of AE‐related drug discontinuation was 17.8% in this study, highlighting that more efforts should be put into AE management during the whole treatment.

This study had some limitations. Due to the retrospective study design, recall bias was inevitable and the frequency of AEs may be underestimated. Some minor discomfort and asymptomatic laboratory abnormalities were difficult to track. In addition, due to the relatively short period of follow‐up, data of overall survival (OS) was not calculated in this study. Although this study proved that prior use of CDK4/6 inhibitors will not affect the efficacy of chidamide, OS data are needed for physicians to optimize the sequence of different strategies in patients progressed on previous endocrine therapies. More prospective studies are needed to address this issue and overcome the challenge of endocrine resistance for patients with luminal breast cancer.

## 5 CONCLUSION

This study provided real‐world data for the use of chidamide in patients with HoR‐positive, HER2‐negative MBC. The efficacy of chidamide was consistent in patients pretreated with CDK4/6 inhibitors and patients treated with different endocrine combinations. Multivariate analysis indicated that patients who had liver metastases (adjusted HR = 1.66, 95% CI 1.14–2.43, adjusted *p* = 0.008) or ≥3 prior lines of treatment (adjusted HR = 1.80, 95% CI 1.17–2.77, adjusted *p* = 0.008) had significantly worse PFS. Further prospective investigations on chidamide with large sample size are warranted in the future.

## AUTHOR CONTRIBUTIONS

**Doudou Li:** Data curation (equal); funding acquisition (equal); investigation (equal); resources (equal); writing – original draft (supporting); writing – review and editing (lead). **Yizi Jin:** Data curation (equal); formal analysis (lead); investigation (equal); visualization (lead); writing – original draft (lead). **Mingxi Lin:** Validation (lead); writing – review and editing (supporting). **Cheng Zeng:** Validation (supporting); writing – review and editing (supporting). **Qing Guo:** Validation (supporting); writing – review and editing (supporting). **Yanfei Liu:** Conceptualization (equal); project administration (equal); supervision (equal). **Jian Zhang:** Conceptualization (equal); funding acquisition (equal); project administration (equal); resources (equal); supervision (equal).

## FUNDING INFORMATION

This study was supported by National Natural Science Foundation of China (grant no. 82072915, 82373359); Project of Shanghai Municipal Health Commission (grant no. 202140397); CSCO‐ROCHE Cancer Research Fund 2019 (grant no. Y‐2019Roche‐171); and Chinese Young Breast Experts Research project (grant no. CYBER‐2021‐001); Beijing Science and Technology Innovation Medical Development Foundation Key Project (grant no. KC2022‐ZZ‐0091‐6); and Shanghai Youth Technological Talent Program—Yangfan Program (grant no. 21YF1408200).

## CONFLICT OF INTEREST STATEMENT

The authors declare that there is no conflict of interest.

## ETHICS STATEMENT

This study was conducted in accordance with the Declaration of Helsinki and approved by Shanghai Cancer Center ethics committees and institutional review boards.

## PATIENT CONSENT STATEMENT

All patients provided written informed consent for the analysis and anonymized publication of clinical data.

## Data Availability

The data that support the findings of this study are available from the corresponding author upon reasonable request.
